# *Cis*,*cis*-muconic acid production from lignin related molecules by*Acinetobacter baylyi* ADP1

**DOI:** 10.1186/s12934-025-02780-3

**Published:** 2025-07-02

**Authors:** Changshuo Liu, Vilja Juvonen, Ella Meriläinen, Elena Efimova, Jin Luo, Milla Salmela, Suvi Santala, Ville Santala

**Affiliations:** Faculty of Engineering and Natural Sciences, Tampere University, Hervanta Campus, PO Box 527, Tampere, FI-33014 Finland

**Keywords:** Protocatechuate decarboxylase, Gallic acid decarboxylase, Lignin related molecules, *cis*, *cis*-muconic acid, Growth-coupled selection, *Acinetobacter baylyi* ADP1

## Abstract

**Background:**

*Cis*,*cis*-muconic acid (ccMA), an important platform chemical, can be produced from lignin related molecules (LRM) via a specific two-branch catabolic route known as the β-ketoadipate pathway, which is present in certain soil bacteria. This pathway enables high production yields because ccMA is a native intermediate in one of its branches. However, commonly obtained LRM, such as *p*-coumaric and ferulic acid, are typically metabolized through the branch that lacks the ccMA intermediate. To redirect these LRM toward ccMA production, the two branches must be functionally integrated. This is usually achieved by introducing a non-native enzymatic activity, specifically protocatechuate decarboxylase (PCADC), which catalyzes the conversion of protocatechuate to catechol. Nevertheless, this conversion often represents the rate-limiting step in the production process.

**Results:**

Here, we established a growth-coupled selection system for screening PCADCs using the soil bacterium *Acinetobacter baylyi* ADP1 as the host. In this system, cell growth depends on the in vivo performance of PCADC, thereby enabling the selection of the optimal candidate for further ccMA production. In total, five PCADC candidates were screened. AGDC1, a gallic acid decarboxylase from the yeast *Blastobotrys adeninivorans*, was selected for production studies. In fed-batch cultivations, the engineered strain expressing AGDC1 achieved an 83% molar yield of ccMA from ferulate and *p*-coumarate, that were found in lignin hydrolysate derived from straw.

**Conclusion:**

In this study, we established a growth-based selection system for PCADCs. The outcome of the selection system was further validated in production cultivations using an engineered *A. baylyi* ADP1 strain. This study not only confirms the feasibility of AGDC1 in ccMA production in bacterial systems but also provides a practical screening system for future improvements.

**Supplementary Information:**

The online version contains supplementary material available at 10.1186/s12934-025-02780-3.

## Background

A bioprivileged molecule is a biology-derived chemical that can be transformed into novel products and drop-in compounds for various industrial applications [1]. A well-known example of a bioprivileged molecule is *cis*,* cis*-muconic acid (ccMA) [1], which can be further converted into multiple products, such as adipic acid (used in nylon production) or terephthalic acid (for polyesters) [2–4]. Several model microorganisms, such as *Escherichia coli*, *Saccharomyces cerevisiae*, and *Pseudomonas putida*, have been engineered for the production of ccMA via either a shikimate or *β*-ketoadipate pathway [3, 5].

The *β*-ketoadipate pathway is a two-branch catabolic pathway found in certain soil bacteria, such as *P. putida* and *Acinetobacter baylyi* ADP1 (later referred to as ADP1), used to catabolize lignin related molecules (LRM) [6, 7]. CcMA is a native intermediate in the catechol branch of the *β*-ketoadipate pathway. This pathway provides an efficient route for production when carbon flux is exclusively funneled to ccMA, offering the potential for equimolar production yields when production is decoupled from growth. This is commonly achieved by blocking the protocatechuate branch and expressing a non-native protocatechuate decarboxylase (PCADC) to convert protocatechuate to catechol. For example, *P. putida* KT2440 strains expressing PCADC have been engineered to produce ccMA from typical LRM such as ferulic and *p*-coumaric acid [8–10]. AroY-type PCADCs, such as those from *Enterobacter cloacae* and *Klebsiella pneumoniae* [11, 12], are prenylated flavine mononucleotide (prFMN)-dependent decarboxylases, which require the iminium form of prFMN as an electron sink for catalysis [12]. To increase the activity of the AroY-type PCADCs, co-expression of proteins responsible for producing the flavin-derived cofactor is commonly needed [9, 13]. Moreover, AroY-type PCADCs have been characterized as oxygen-sensitive enzymes, their half-life being only 5–8 min under aerobic conditions [12]. A recently characterized gallic acid decarboxylase AGDC1 from *Blastobotrys adeninivorans* [14, 15] was found to also catalyze the conversion of protocatechuate to catechol [16]. Interestingly, AGDC1 is reported to be a cofactor-independent decarboxylase [15].

Growth-coupled selection is an efficient approach for enzyme or pathway screening by associating cell growth with the in vivo performance of a target enzyme. Growth-coupling can be achieved by strategically interrupting metabolic pathways to make growth dependent on the function of the target [17–19]. The approach enables high-throughput screening without the need for complex assays, providing an efficient way to test non-native enzymes or novel enzyme variants. Numerous applications of the growth-coupled selection approach have been previously described, for example, the screening of heterologous methyltransferases in *S. cerevisiae* [20].

In this study, we established a growth-coupled selection platform for screening several PCADC activities using ADP1 as the host. ADP1 possesses the well-characterized *β*-ketoadipate pathway [6] and, more importantly, can efficiently utilize LRM from various streams [21, 22]. Additionally, ADP1 has highly efficient natural transformation and homologous recombination machinery, allowing for easy and rapid genome engineering [23]. Based on these attributes, ADP1 has the potential to serve as a platform for lignin valorization [24–27]. By employing the PCADC selected through the established method, we demonstrate the production of ccMA from typical LRM, ferulic acid and *p*-coumaric acid, using engineered ADP1.

## Methods

### Strains

A wild-type ADP1 strain (DSM 24193, DSMZ, Germany) and its derived strains were used for the growth-coupled selections and ccMA production. *Escherichiacoli* XL1-Blue (Stratagene, USA) was used as a host for plasmid constructions. The strains used in this study are listed in Table 1.

**Table 1:** *Strains used in this study.*

Strain designation	Description	Reference
ADP1	Wild-type ADP1	DSM 24193, DSMZ
XL1	*E.coli* XL1-Blue	Stratagene, USA
ASA901	ADP1; Δ*pcaHG*::*tdk/kan*^*R*^	This study
ASA902	ADP1; Δ*pcaHG*	This study
ASA903	ASA901; Δ*poxB*::*P*_*t5*_*-AroY_kp-cm*^*R*^	This study
ASA904	ASA901; Δ*poxB*::*P*_*t5*_*-AroY_ph-cm*^*R*^	This study
ASA905	ASA901; Δ*poxB*::*P*_*t5*_*-AroY_ks-cm*^*R*^	This study
ASA906	ASA901; Δ*poxB*::*P*_*t5*_*-AroY_au-cm*^*R*^	This study
ASA907	ASA901; Δ*poxB*::*P*_*t5*_*-ADGC1-cm*^*R*^	This study
ASA915	ASA902; Δ*catMBC; ΔbenR*	This study
ASA916	ASA915; Δ*poxB*::*P*_*t5*_*-AGDC1-cm*^*R*^	This study
ASA917	ASA916; pBAV1k*-lacI-*P_trc_*-*BCD9-catA*	This study

### Media and cultivation conditions

ADP1 and XL1 cells were grown for cloning and transformation purposes in 14 mL culture tubes with 5 mL low salt LB medium (tryptone 10 g/L, yeast extract 5 g/L, NaCl 1 g/L), or LB agar plates (adding 15 g/L agar to the low salt LB medium) supplemented with 50 mM glucose and the appropriate antibiotics (chloramphenicol 25 µg/mL, kanamycin 30 µg/mL). The liquid cultivations were incubated at 30 °C and 300 rpm, cultivations using LB agar plates were incubated at 30 °C. For the *tdk/kan*^*R*^ rescue screening purpose, the solid medium was supplemented with 400 µg/mL azidothymidine.

ADP1 precultures for strain characterization and production cultivations were inoculated from LB agar plates. Main cultivations were carried out using mineral salts medium (MSM) [28] supplemented with different carbon sources. The composition of the MSM was as follows: K_2_HPO_4_ 3.88 g/L, NaH_2_PO_4_ 1.63 g/L, (NH_4_)_2_SO_4_ 2.00 g/L, MgCl_2_ ∙ 6H_2_O 0.1 g/L, ethylenediaminetetraacetic acid 10 mg/L, ZnSO_4_ ∙ 7H_2_O 2 mg/L, CaCl_2_ ∙ 2H_2_O 1 mg/L, FeSO_4_ ∙ 7H_2_O 5 mg/L, Na_2_MoO_4_ ∙ 2H_2_O 0.2 mg/L, CuSO_4_ ∙ 5H_2_O 0.2 mg/L, CoCl_2_ ∙ 6H_2_O 0.4 mg/L, MnCl_2_ ∙ 2H_2_O 1 mg/L.

For the growth-coupled selections, the cells were precultivated in 5 mL LB medium in 14 mL culture tubes at 30 °C and 300 rpm overnight. Cells were collected and washed three times from the overnight precultures were used to inoculate 200 µL MSM supplemented with 10 mM 4-hydroxybenzoate in a 96-well plate, cultivated using Spark multimode microplate reader (Tecan, Switzerland) at 30 °C.

To compare the ccMA production of ASA916 and ASA917, the cells were precultivated in 5 mL MSM supplemented with 1 mM 4-hydroxybenzoate, 50 mM gluconate, 0.4% (w/v*)* casein amino acids, and 25 µg/mL chloramphenicol in 14 mL culture tubes at 30 °C and 300 rpm for overnight. Cells collected from the overnight precultures were used to inoculate 15 mL MSM supplemented with 50 mM gluconate, 0.4% (w/v*)* casein amino acids, 2 mM or 5 mM coumarate, and 25 µg/mL chloramphenicol in 50 mL tubes at 30 °C and 300 rpm. Isopropyl β-D-1-thiogalactopyranoside (IPTG) at a final concentration of 100 µM was added to the ASA917 cultivations 2 h after inoculation.

Fed-batch cultivations were carried out in a 250 mL mini bioreactor (Applikon Biotechnology, Netherlands). The cells were precultivated in 5 mL MSM supplemented with 0.5 mM *p*-coumarate, 0.5 mM ferulate, 50 mM gluconate, 0.2% (w/v) casein amino acids, 100 µM IPTG, and 30 µg/mL kanamycin in 14 mL culture tubes at 30 °C and 300 rpm overnight. The precultures were then inoculated into 50 mL of the same medium in 250 mL Erlenmeyer flasks at 30 °C and 300 rpm overnight. The cells were collected and resuspended into 50 mL fresh medium in the bioreactor, the cultivations were conducted at 30 °C and agitation 200 rpm, the culture pH was maintained at 7.0 using 5 M H_3_PO_4_. From 0 h to 28 h, MSM supplemented with 2.5 mM *p*-coumarate, 2.5 mM ferulate, 50 mM gluconate, 0.2% (w/v) casein amino acids, 100 µM IPTG, and 30 µg/mL kanamycin was fed to the bioreactor with flow rate of 3.5 mL/h. From 28 h to 54 h, MSM supplemented with 50 mM gluconate, 0.2% (w/v) casein amino acids, 100 µM IPTG and 30 µg/mL kanamycin was fed to the bioreactor with the same flow rate.

### Strain construction

The transformation and homologous recombination-based genome editing of ADP1 were done as described by Santala et al. [29]. Wild-type ADP1 was used as the parental strain. The *tdk/kan*^*R*^ cassette was amplified with MfeI and AvrII restriction sites from the genome of ADP1 Δ*acr1*::*tdk/kan*^*R*^ [30] (a kind gift from Veronique de Berardinis, Genoscope, France). A plasmid pUC57-Δ*pcaHG* containing flanking for targeted knock-out was purchased from GenScript (Netherlands). The plasmid and the *tdk/kan*^*R*^ cassette were digested using MfeI and AvrII, and then ligated to generate a plasmid pUC57-Δ*pcaHG*::*tdk/kan*^*R*^. ASA901 was obtained by transforming the plasmid pUC57-Δ*pcaHG*::*tdk/kan*^*R*^ into wild-type ADP1, and ASA902 was obtained by rescue using plasmid pUC57-Δ*pcaHG*. The codon-optimized genes *aroY_kp*, *aroY_ph*, *aroY_ks*, *aroY_au*, and *AGDC1* were purchased from GenScript (Netherlands). These genes were digested using NdeI and XhoI, and then ligated to the previously constructed i-cassette which contains flankings for *poxB* (ACIAD3381) gene knock-out, T5 promoter, and a marker for chloramphenicol selection (*cm*^*R*^) [31]. ASA903-ASA907 strains were obtained by transforming these gene cassettes into ASA901. ASA915 was constructed first deleting *catMBC* genes by *tdk/kan*^*R*^ knock-out cassette and rescuing as above. Then *benR* gene was deleted similarly. ASA916 was constructed by transforming AGDC1 overexpression cassette, i-P_t5_*-AGDC1-cm*^*R*^, into ASA915. To construct the strain with the overexpression of *catA*, a plasmid pBAV1k-*lcaI-*Ptrc*-*BCD9-catA* (supplementary Figure S1) was constructed using Gibson assembly. For that, the pBWB294 (a gift from Keith Tyo, Addgene #140636) plasmid backbone [32] was obtained from Addgene (USA) and a gene fragment for codon-optimized *catA* (catA*) was obtained from GenScript (Netherlands). The overexpression plasmid was transformed into ASA916 to obtain ASA917. All constructed strains were confirmed by PCR. PCR products and plasmids were sequenced (Macrogen, Netherlands). The primers and PCR reagents were obtained from Thermo Scientific (USA). The primers, plasmids, and relevant gene sequences used in this study are listed in the supplementary Table S1, S2, and S3, respectively.

### Analytical methods

Substrates, relevant intermediates, and muconates (*cis*,* cis*-muconate and *cis*,* trans*-muconate) were analyzed by high-performance liquid chromatography (HPLC). Muconates, ferulate, *p*-coumarate, protocatechuate, catechol, benzoate, vanillate, and 4-hydroxybenzoate were detected with an HPLC device (LC-40D, Shimadzu, Japan) equipped with a photodiode array detector (SPD-M40, Shimadzu, Japan). A C18 reverse-phase column (Luna, 5 μm particle size, 4.6 × 150 mm; Phenomenex, USA) was used and maintained at 40 °C. A mixture of water: methanol: formic acid (80: 20: 0.16, v/v/v) was used as mobile phase with the flow rate of 1.0 mL/min. *Cis*,* cis*-muconate and *cis*,* trans*-muconate standards were prepared using the method described by Black et al. [33]. Gluconate was analyzed with an HPLC device (LC-20AC, Shimadzu, Japan) equipped with a refractive Index detector (RID-10 A, Shimadzu, Japan) on a Rezex RHM-monosaccharide H+ (8%) column (7.8 × 300 mm; Phenomenex, USA) maintained at 70 °C. Sulfuric acid (0.01 N) was used as mobile phase with the flow rate 0.6 mL/min.

### Results and discussion

The *β*-ketoadipate pathway provides an efficient route for producing ccMA from LRM, as ccMA represents a direct intermediate in the catabolic pathway. However, the protocatechuate and catechol branches of the *β*-ketoadipate pathway are not naturally connected, thereby preventing the production of ccMA from the most common monomeric compounds found in depolymerized lignin, namely *p*-coumaric acid and ferulic acid [34]. To connect the branches and to allow the monomers to funnel to ccMA, non-native PCADCs converting protocatechuate to catechol can be expressed [8, 11]. For example, AroY-type PCADCs have been widely used [8–10]. However, this non-native conversion step has turned out to be inefficient due to low catalytic rates and poor oxygen tolerance of PCADCs [13, 35]. Although co-expression of cofactor-regenerating enzymes has been reported to improve the activity of AroY-type PCADC [9, 13], exploring alternative types of PCADCs could offer a potential solution.

### Growth-coupled selection system

**Fig. 1:**
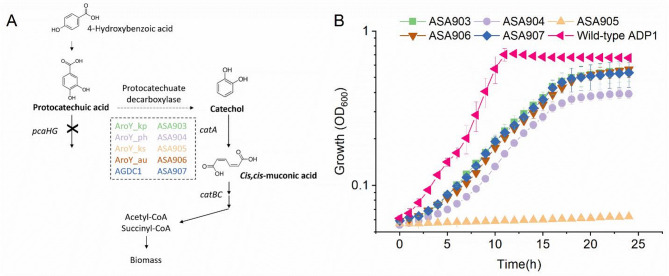
**(A)** Modified metabolic pathway for the growth-coupled selection of the PCADC candidates. Arrow with solid line indicates native pathway; arrow with dashed line indicates heterologous activity; X indicates pathway blocked by the gene deletions. **(B)** Growth of ASA903, ASA904, ASA905, ASA906, ASA907, wild-type ADP1 in MSM supplemented with 10 mM 4-hydroxybenzoate as a sole carbon source. The experiment was repeated using independent biological triplicates. The averages of the measurements, with error bars representing standard deviations are shown. The y-axis is shown on the log_10_ scale

To screen potential PCADC candidates, we first set up a growth-based selection system using ADP1 as a host. The system enables straightforward screening of multiple enzyme candidates as cell growth can be linked to the in vivo performance of the enzymes. To couple the in vivo performance of PCADC with growth, we first deleted the *pcaHG* genes in the protocatechuate branch of the *β*-ketoadipate pathway of ADP1 (Fig. 1A). The resulting strain, ASA901, cannot utilize *p*-coumaric acid or ferulic acid as a carbon source for cell growth (data not shown), unlike the wild-type strain. The gallic acid decarboxylase AGDC1 was of interest to us because it is a cofactor independent decarboxylase [15]. In addition to AGDC1, we also tested several AroY-type PCADCs from other species that were similar in amino acid sequence to that from *K. pneumonia* [12]. Using the amino acid sequence of AroY from *Klebsiella pneumonia* (AroY-kp) as a reference, four PCADCs with similar amino acid sequences were selected as candidates using the Basic Local Alignment Search Tool (BLAST): AroY-ph (from *Pedobacter himalayensis*), AroY-ks (from *Kosakonia* sp. MUSA4), and AroY-au (*Actinomyces urogenitalis* S6-C4) (Supplementary Table S4). For the overexpression, the codon-optimized genes of the candidate decarboxylases were cloned to a gene cassette and integrated to the genome of ASA901 to obtain strains ASA903-907, respectively.

The growth-coupled selection was carried out in 96-well plate liquid cultivations (Fig. 1B). A concentration of 10 mM 4-hydroxybenzoate was used, as it supported sufficient growth as the sole carbon source without any observable toxicity. ASA903 (with AroY-kp), ASA906 (with AroY-au), and ASA907 (with AGDC1) showed similar growth performances. ASA904 (with AroY-ph) showed a somewhat longer lag phase, and ASA905 (with AroY-ks) did not grow under the studied conditions. The selection cultivation was also conducted using a solid medium (supplementary Figure S2), which exhibited similar results to the liquid cultivation.

According to the results, AGDC1, AroY-kp, and AroY-au exhibited the highest in vivo performance among all PCADCs tested, demonstrating that AroY-type PCADCs and AGDC1 are functional in ADP1. For the subsequent experiments and the construction of the production strain, AGDC1 was selected due to its cofactor independence and novelty in the context of bacterial production from LRM. Although AroY-type PCADCs have been shown to be functional in *P. putida* KT2440 without additional cofactor biosynthesis, co-expression of cofactor-supplying genes is often employed to enhance their catalytic efficiency and product yield [9, 13]. In contrast, AGDC1 does not require such supplementation, offering a simpler and potentially more efficient solution for pathway design with a reduced number of non-native proteins. Additionally, AGDC1 has been previously employed in ccMA production via a modified shikimic acid pathway in *S. cerevisiae*, providing improved yields compared to AroY from *K. pneumoniae* [36].

### Engineering ADP1 for *cis*,*cis*-muconic acid production

After the selection, ADP1 was further engineered for production purposes, i.e. ccMA production was decoupled from the catabolic pathway. For this, the genes *catBC*, which encode muconate cycloisomerase and muconolactone D-isomerase, respectively, were deleted to prevent ccMA utilization. To verify the deletion and functionality of the engineered strain for ccMA production, the ADP1 strain with *catBC*-deletion was batch-cultivated in a medium supplemented with benzoate. Approximately 100% of the consumed benzoate was converted to ccMA, which can be expected due to ccMA being a direct catabolic intermediate in the benzoate utilization pathway (supplementary Figure S3). The production of ccMA from benzoate via the *β*-ketoadipate pathway has been reported in *Arthrobacter* and *Pseudomonas* strains, with molar yields up to 96 % [37, 38].

In ADP1, transcriptional regulators CatM and BenR have been reported to respond to ccMA and repress the protocatechuate branch [39]. In addition, it was previously reported that the expression of AroY in *P. putida* to produce ccMA from e.g. 4-hydroxybenzoate resulted in low conversion rate and accumulation of intermediates [8]. Thus, to prevent the repression, we deleted the genes *catM* and *benR* in the production strain exhibiting the deletions *pcaHG* and *catBC*, in addition to the overexpression of AGDC1. The obtained strain was designated as ASA916. The ccMA production of ASA916 was evaluated by fed-batch cultivation with 4-hydroxybenzoate as the substrate (supplementary Figure S4). The cells produced 8.8 mg ccMA from 22.3 mg 4-hydroxybenzoate (yield 0.39 g/g). Accumulation of protocatechuate was observed in the culture, of which part remained until the end of the cultivation, explaining the suboptimal yield of ccMA.

**Fig. 2:**
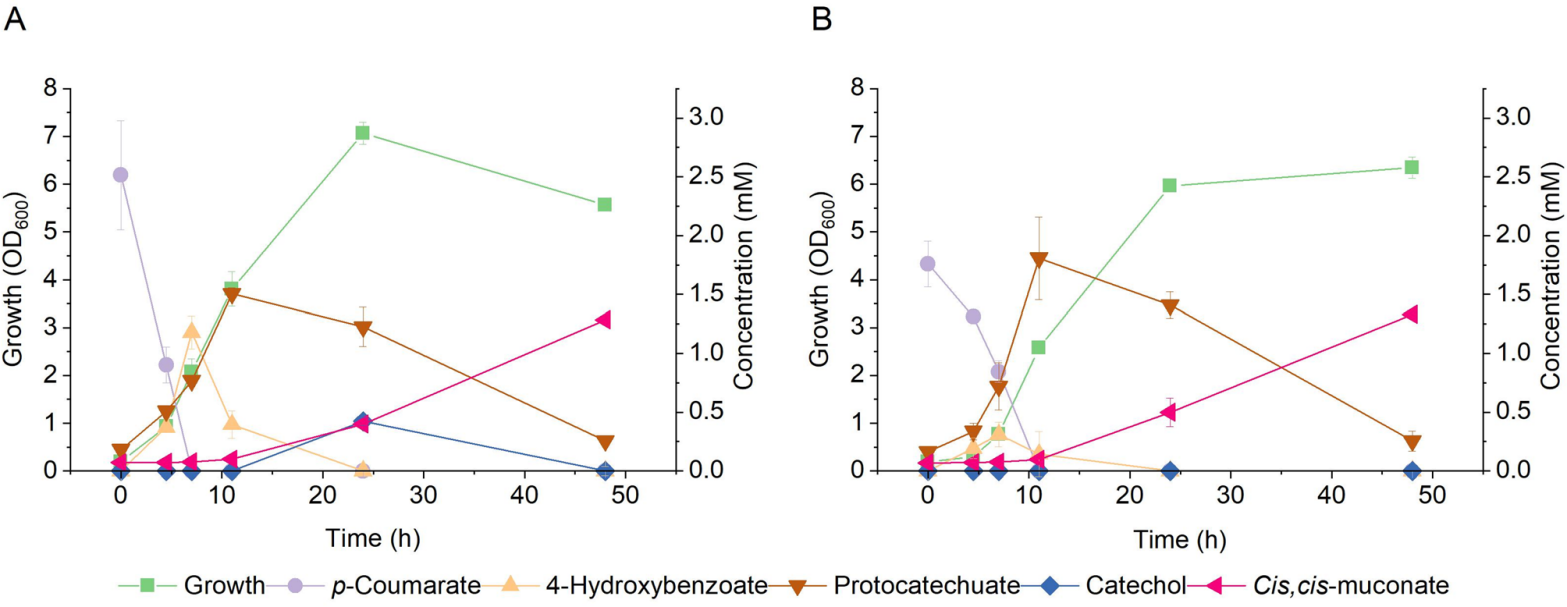
Growth and ccMA production kinetics of **(A)** ASA916 and **(B)** ASA917 in batch cultivations with *p*-coumarate. The experiment was repeated using independent biological triplicates, and the averages of the measurements, with error bars representing standard deviations are shown

Protocatechuate and catechol were both found to accumulate in the cultures during the production of ccMA by ASA916 (Fig. 2A). Thus, we hypothesized that overexpressing the *catA* gene (encoding catechol 1,2-dioxygenase) could improve ccMA production, as several previous studies have demonstrated a positive effect of *catA* overexpression on ccMA yield [37, 40]. To this end, an overexpression plasmid carrying the *catA* gene was transformed into ASA916 strain, resulting in a new strain designated as ASA917. A batch cultivation was conducted to compare ccMA production of ASA917 (Fig. 2B) with that of ASA916 (Fig. 2A). As expected, both ASA916 and ASA917 produced ccMA from *p-*coumaric acid. In line with previous studies using other bacteria [37, 40], the overexpression of *catA* improved the yield. The ccMA yield was 21.2 ± 3.5% from 2 mM *p*-coumarate with the ASA916 strain, while 40.5 ± 2.4% yield was obtained using the ASA917 strain. Accumulation of catechol in ASA916 cultures at 24 h, contrasted with its absence in the cultivations of the *catA*-overexpressing strain ASA917, suggests that *catA* overexpression enhances the conversion of catechol to ccMA.

### *Cis*,*cis*-muconic acid production from *p*-coumarate and ferulate

To further demonstrate the production of ccMA from relevant substrates we conducted fed-batch cultivations with ASA917 strain (supplementary Figure S5). To identify the relevant aromatics and their concentrations for the cultivations, we analyzed the hydrolysate of straw-derived organosolv lignin (a kind gift from Fortum, Finland). The lignin hydrolysate contained approximately 2.0 mM ferulic acid and 2.2 mM *p*-coumaric acid (Supplementary Figure S6).

**Fig. 3:**
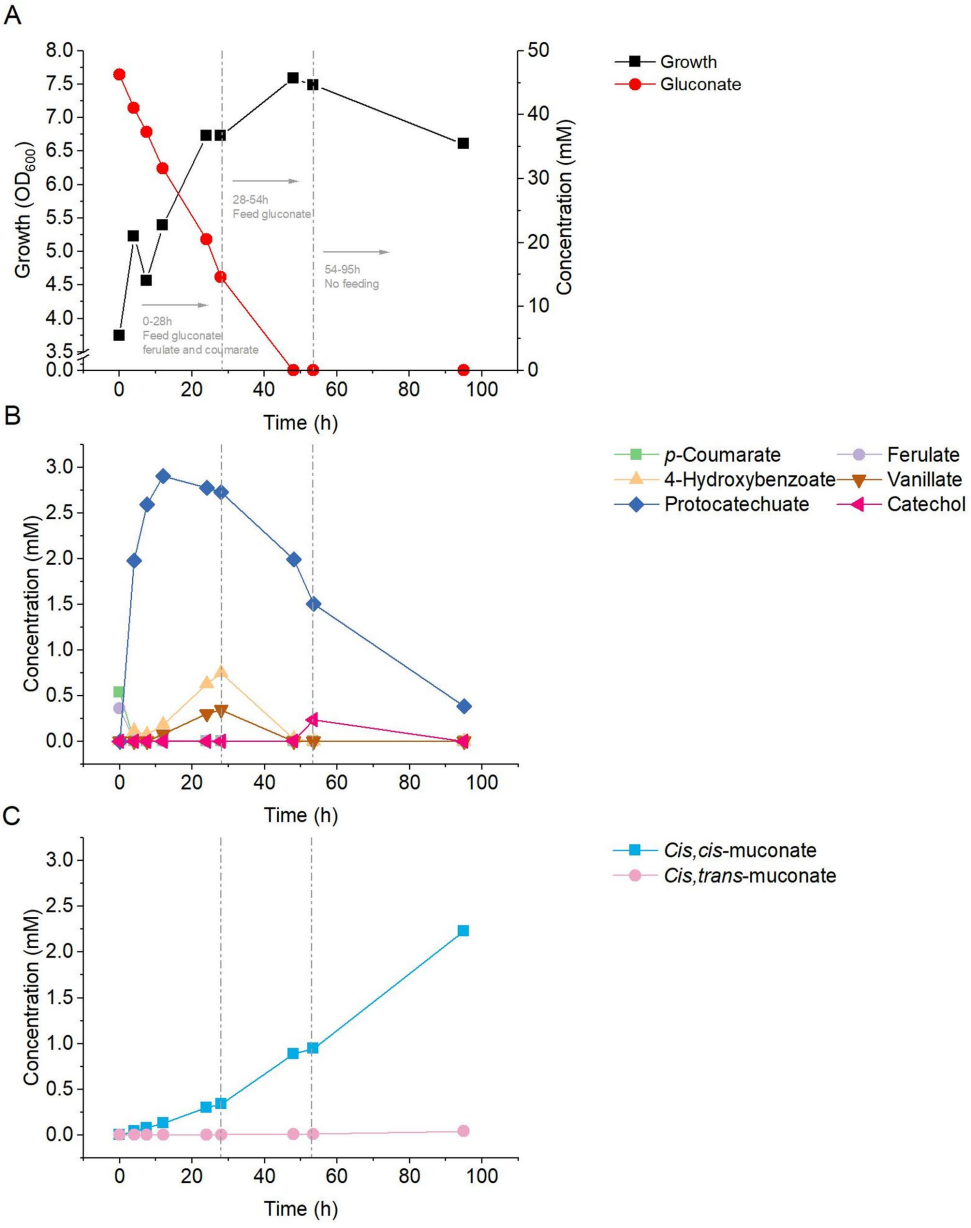
Growth of ASA917 and the kinetics of substrate utilization and product formation. **(A)** growth and gluconate, **(B)** ferulate, *p*-coumarate, and intermediates (vanillate, 4-hydroxybenzoate, protocatechuate, catechol), and **(C)** products (*cis*,*cis*- and *cis*,*trans*-muconate)

We then set up a fed batch cultivation for the engineered strain. To account for batch-to-batch variability and simplify process parameters, a mixture containing equal amounts of both acids (2.5 mM) was used as a substrate for cultivation, with gluconate supplied as an additional carbon and energy source to support cell growth. We first carried out a cultivation, where feeding was terminated at 48 h time point (supplementary Figure S7). After 95 h, 70% yield of ccMA from consumed substrates, 0.53 mmol *p*-coumaric acid and 0.36 mmol ferulic acid, was obtained. During cultivation, only protocatechuate accumulated during substrate feeding. This accumulation was significant within the first 28 h, when substrate feeding was ongoing. At this stage, approximately 0.69 mM of ccMA was produced, accounting for only 20% of the final ccMA titer (3.43 mM). After 28 h, the concentration of protocatechuate began to decline gradually and then decreased rapidly following the cessation of substrate feeding at 48 h. Thus, for the next run, longer feeding period of gluconate (0–54 h) and shorter period for aromatics (0–28 h) were tested (Fig. 3 and supplementary Figure S7). The additional gluconate feeding was hypothesized to provide more ATP for the cells, which could support the conversion of catechol to ccMA during the cultivation [41]. The produced ccMA remained in *cis*,* cis*-form, and only a trace amount (0.01 mmol) of *cis*,* trans*-form, typically appearing in conditions with pH lower than 7 [33], was detected at some sampling points. Ferulate and *p*-coumarate were only observed at the start of the cultivation; low concentrations of the intermediates (vanillate, 4-hydroxybenzoate, protocatechuate, and catechol) were detected during the cultivation (Fig. 3B). Except for approximately 0.07 mmol protocatechuate, no other intermediates were detected at the end of the cultivation.

A total 0.32 mmol *p-*coumaric and 0.2 mmol ferulic acids were converted to 0.43 mmol of ccMA in a 95-h cultivation, providing a yield of 83.4%. With *P. putida*, Salvachúa et al. reported a 100% molar yield ccMA by removing the global regulator of carbon catabolite repression and enhancing the activity of the PCADC [10]. Thus, there is potential to further improve production in ADP1. Accumulation of protocatechuate has been previously identified as a major bottleneck in ccMA production [8, 9], which was also the case in our process. Although *catA* was overexpressed in ASA917 to improve yields, it did not have pronounced effect on overcoming protocatechuate accumulation. It should be noted that compared with CatA of *P. putida*, ADP1’s native CatA exhibits lower activity [42]. However, a proline to alanine mutation at position 76 of ADP1’s CatA has been reported to improve its activity 10-fold [42]. Another potential strategy to enhance production is to improve the AGDC1 enzyme through directed evolution. Combining our previously introduced rapid mutation screening and selection method (RAMSES) [43] with the growth-coupled selection could provide an efficient system for screening AGDC1 variants.

## Conclusion

In this study, our aim was to establish a growth-coupled selection method to identify potential candidate enzymes for the key step in ccMA production from LRM. We utilized a native LRM-utilizing strain *A. baylyi* ADP1 as the host. Based on the growth results obtained in this study and previously reported properties, the gallic acid decarboxylase AGDC1 from *B. adeninivorans* was selected for the construction of a ccMA-producing strain. Through metabolic engineering, 83.4% molar yield of ccMA was achieved by the engineered ADP1. This work demonstrates the potential and feasibility of AGDC1 for bacterial ccMA production and highlights the promise of further improvements using the growth-coupled selection method.

## Electronic supplementary material

Below is the link to the electronic supplementary material.

Supplementary Material 1

## Data Availability

The data that support the findings of this study are included within the article or the additional files. The corresponding author is willing to provide the raw data related to this manuscript upon reasonable request.
