# Supplementary material for: Cis,cis-muconic acid production from lignin related molecules byAcinetobacter baylyi ADP1
Source: Microb Cell Fact. 2025 Jul 2;24:150. doi: 10.1186/s12934-025-02780-3 (PMC12220188; doi:10.1186/s12934-025-02780-3)
Supplement: Supplementary file 1 — Supplementary Material 1 [file 12934_2025_2780_MOESM1_ESM.docx]

**Supplementary information**

***Cis,cis*-muconic acid production from lignin related molecules by *Acinetobacter baylyi* ADP1**

Changshuo Liu ^1^, Vilja Juvonen ^1^, Ella Meriläinen ^1^, Elena Efimova ^1^, Jin Luo ^1^, Milla Salmela ^1^, Suvi Santala ^1^, and Ville Santala ^1*^

^1^ Faculty of Engineering and Natural Sciences, Hervanta Campus, Tampere University, PO Box 527, FI-33014 Tampere, Finland

^*^Corresponding author: Ville Santala (ville.santala@tuni.fi)


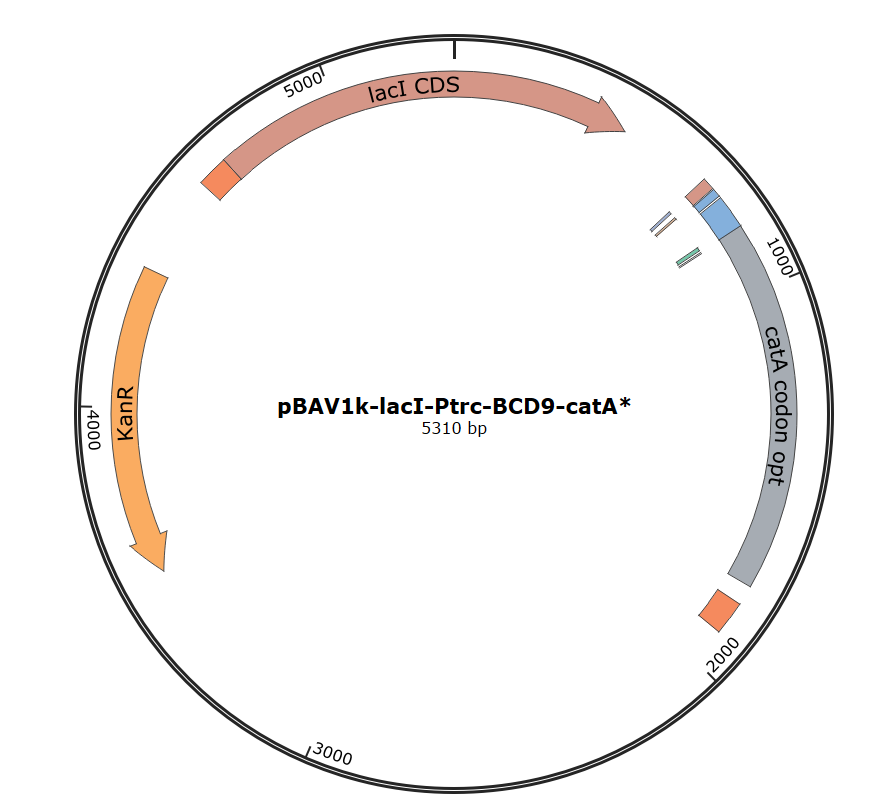


**Figure S1.** Plasmid map of pBAV1k-*lacI-*Ptrc*-*BCD9*-catA**.

**Table S1.** Primers used in this study.

| **Purpose** | **Template** | **ID** | **Description** | **Sequences (5’ to 3’)** |
| --- | --- | --- | --- | --- |
| Amplification of the genes *tdk/kan^R^* with restriction sties MfeI and AvrII | ADP1 *Δacr1*::*tdk/kanR* [1] | VS_21_01 | FW | TAATACAATTGCCCAGCCTCCAATTCAAATC |
|  |  | VS_21_02 | RV | TAATACCTAGGCCAGCTCCGCATGCTTAG |
| Verification of *pcaHG* knock-out | ASA901 and ASA902 | KO-1711-1712-vp1 | FW | TGTTAAATCCCTTGTTGCAC |
|  |  | KO-1711-1712-vp2 | RV | CAATACTTTTTTCTGGTGCG |
| Verification of *poxB* knock-out | ASA903-ASA907, ASA916 | 3381v1 | FW | TTGGCTAACTTGTCAAAGTC |
|  |  | 3381v2 | RV | TAGAGTGTAGAAACAGATGC |
| Amplification of the genes *tdk/kan^R^* for the construction of knockout cassettes via using SOE PCR | ADP1 *Δacr1*::*tdk/kanR* [1] | tdkkan F ko | FW | CCCAGCCTCCAATTCAAATCATAAAAAATTTATTTG |
|  |  | tdkkan R ko | RV | CCAGCTCCGCATGCTTAGAAAAAC |
| Amplification of the upstream flanking sequence for *catBCM* knock-out (contains an overhang sequence for *tdk/kan^R^*) using SOE PCR | ADP1 | CatM_P5 | FW | TTTTTATGATTTGAATTGGAGGCTGGGAATATGTCTGAAAAATTTACTGC |
|  |  | CatM_P6 | RV | TTGGATTCGCTTAGCAAGATCG |
| Amplification of the downstream flanking sequence for *catBCM* knock-out, (contains an overhang sequence for *tdk/kan^R^*) using SOE PCR | ADP1 | CatC_P5 | FW | CGATGAGTTTTTCTAAGCATGCGGAGCTGGTATGTAAATAGAGATGACACTTC |
|  |  | CatC_P6 | RV | CCATAGTCTCCATTGCCGGC |
| Amplification of the upstream flanking sequence for *catBCM* markerless rescue cassette (contains MfeI, NotI, AvrII) using SOE PCR | ADP1 | CatM_rescueP5 | FW | CCTAGGGCGGCCGCCAATTGAATATGTCTGAAAAATTTACTGC |
|  |  | CatM_P6 | RV | TTGGATTCGCTTAGCAAGATCG |
| Amplification of the downstream flanking sequence for *catBCM* markerless rescue cassette (contains MfeI, NotI, AvrII) using SOE PCR | ADP1 | CatC_rescueP5 | FW | CAATTGGCGGCCGCCCTAGGTATGTAAATAGAGATGACACTTC |
|  |  | CatC_P6 | RV | CCATAGTCTCCATTGCCGGC |
| Amplification of the upstream flanking sequence for *benR* knock-out (contains an overhang sequence for *tdk/kan^R^*) using SOE PCR | ADP1 | BenR_P3 | FW | CATATCCTTTCTCCAACATTCC |
|  |  | BenR_P4 | RV | TTTTTATGATTTGAATTGGAGGCTGGGTTAAAAATACTCCATAGGTATTT |
| Amplification of the downstream flanking sequence for *benR* knock-out, (contains an overhang sequence for *tdk/kan^R^*) using SOE PCR | ADP1 | BenR_P5 | FW | CGATGAGTTTTTCTAAGCATGCGGAGCTGGCAAAAAACCCATCAGACTGTG |
|  |  | BenR_P6 | RV | CTACTGGAATTGGTTGGGGGC |
| Amplification of the upstream flanking sequence for *benR* markerless rescue cassette (contains MfeI, NotI, AvrII) using SOE PCR | ADP1 | BenR_P3 | FW | CATATCCTTTCTCCAACATTCC |
|  |  | BenR_rescueP4 | RV | CAATTGGCGGCCGCCCTAGGTTAAAAATACTCCATAGGTATTT |
| Amplification of the downstream flanking sequence for *benR* markerless rescue cassette (contains MfeI, NotI, AvrII) using SOE PCR | ADP1 | BenR_rescueP5 | FW | CCTAGGGCGGCCGCCAATTGCAAAAAACCCATCAGACTGTG |
|  |  | BenR_P6 | RV | CTACTGGAATTGGTTGGGGGC |
| Verification of *catMBC* knock-out | ASA915 |  | FW | GATCATTTTTTTGGCTTCGGC |
|  |  |  | RV | TGCGGCACGATTTCAAGTTC |
| Verification of *benR* knock-out | ASA916 |  | FW | TCGCTTGGATCTTTCACCTTC |
|  |  |  | RV | CTGGGTGCGATTTCACTGAG |
| Amplification of the codon optimized *catA* for Gibson assembly | pUC57-*catA** | JL22-5 | FW | AGTCTTTCTAATGGAAGTAAAAATTTTTAATACTCAAGATGTTCAAG |
|  |  | JL22-6 | RV | GCCCTGAGGCTTACACTGCTAAACGAGGACGAT |
| Amplification of the plasmid backbone of pBWB294 for Gibson assembly | pBWB294 [2] | JL22-7 | FW | AGCAGTGTAAGCCTCAGGGCCCGATC |
|  |  | JL22-8 | RV | AATTTTTACTTCCATTAGAAAGACTCCTCTGCATGATTAAGATG |

**Table S2.** Plasmids used in this study.

| **Plasmid** | **Description** | **Antibiotic resistance** | **Source** |
| --- | --- | --- | --- |
| pUC57-Δ*pcaHG* | Knock-out cassette of *pcaHG* | AmpR | This study |
| pUC57-Δ*pcaHG*::*tdk/kan^R^* | Knock-out cassette of *pcaHG* with *tdk/kan^R^* | AmpR, KanR | This study |
| i/pIX | Knock-out cassette of *poxB* | CmR | [3] |
| i-P_t5_*- aroY_kp-cm^R^*/pIX | Knock-out cassette of *poxB* with decarboxylase coding gene | CmR | This study |
| i-P_t5_*- aroY_ph-cm^R^*/pIX | Knock-out cassette of *poxB* with decarboxylase coding gene | CmR | This study |
| i-P_t5_*- aroY_ks-cm^R^*/pIX | Knock-out cassette of *poxB* with decarboxylase coding gene | CmR | This study |
| i-P_t5_*- aroY_au-cm^R^*/pIX | Knock-out cassette of *poxB* with decarboxylase coding gene | CmR | This study |
| i-P_t5_*-AGDC1-cm^R^*/pIX | Knock-out cassette of *poxB* with decarboxylase coding gene | CmR | This study |
| pBAV1k-*lacI-*Ptrc*-*BCD9*-catA** | Overexpression cassette of codon optimized *catA* | KanR | This study |
| pBWB294 | The source of the pBAV1k backbone with a trc promoter and a BCD9 ribosome binding site | KanR | [2] |
| pUC57-*catA** | Plasmid with a codon optimized *catA* gene | AmpR | This study |

**Table S3.** Codon optimized synthetic genes used in this study.

| **Enzyme** | **Coding sequence (5’ to 3’)** |
| --- | --- |
| AroY_kp | atgacagcaccaattcaagatttacgtgatgctattgcattattgcaacagcatgataatcagtatcttgaaaccgatcatccagttgatcctaatgctgaactggcaggtgtgtatcgtcatattggtgcaggtggtacagtaaaacgtcctacccgtattggtccagcaatgatgtttaataatattaaaggttatccacattctcgtattttagtcggtatgcatgcaagccgtcaacgtgcagctcttctgttaggttgtgaagctagtcagctggcattagaagttggtaaagctgtgaaaaaaccagtagcacctgttgttgttccagctagttcagcaccatgtcaagaacagatttttcttgcagatgatccagattttgatttgcgtacattgcttccagctcctactaatacacctattgatgcaggtccatttttctgtttaggtcttgcactggctagtgatcctgtggatgcatcacttaccgatgtaactattcatcgtctgtgtgtccaaggtcgtgatgaactttcaatgtttctggcagctggtcgtcatattgaagtttttcgtcagaaagcagaagcagctggtaaaccactgcctattaccattaatatgggtttagatccagcaatttatattggtgcttgttttgaagcaccaacaaccccttttggttataatgaacttggtgtggcaggtgctctgcgtcaacgtcctgtcgaattagttcagggtgtctctgttccagaaaaagctattgcacgtgcagaaattgtaattgaaggtgaactgttacctggtgtgcgtgtacgtgaagatcaacatacaaatagtggtcatgctatgccagaatttcctggttattgtggtggtgcaaatccatcactgcctgtgattaaagtaaaagctgtcaccatgcgtaataatgcaattttacaaactttggttggtcctggtgaagaacatactacacttgcaggtctgccaactgaagctagtatttggaatgcagtagaagcagctattccaggttttcttcaaaatgtctatgctcatacagcaggtggtggtaaatttttaggtattttgcaagtgaaaaaacgtcagccagcagatgaaggtcgtcagggtcaggcagctttgcttgctttggcaacctattctgaactgaaaaatattattttggtcgatgaagatgttgatatttttgatagcgatgatattctgtgggcaatgaccactcgtatgcaaggtgatgttagtattacaaccattccaggtattcgtggtcatcaattagatccatcacagactcctgaatattctccaagcattcgtggtaatggtatttcttgtaaaactatttttgattgtacagttccttgggcacttaaaagccattttgaacgtgctccatttgcagatgttgatccacgtccttttgctcctgaatattttgcacgtttagagaaaaatcaaggttcagcaaaataa |
| AroY_ph | atgaaaaaacatattaatgatttacgtagtgctattgaactgttgaaacgtcatgaaggtcaatatttagaaacatcacatccagttgatcctgatgctgaattggcaggtgtgtatcgtcatattggtgcaggtggtactgtgaaacgtcctacacgtattggtccagctatgatgtttaatgcaattaaaggttatccagatagtcgtattcttgtaggcatgcatgcatcacgtgaacgtgcagctcttctgttaggttgtgaacctagtgaattagctaaacatgtaggtcaagcagtcaaaaatcctattgcacctgttgttgttccagcagcacaggcaccatgtcaagaacaggtgttttatgcagatgatccagattttgatttgcgtaaattgcttccagcacctacaaatacccctattgatgctggtccatttttctgtttaggtttagttctggcatctgatccagaagatgctagccttaccgatgtaactattcatcgtctgtgtgtccaagaacgtgatgaattatcaatgtttttggcagctggtcgtcatattgaagtttttcgtaagaaagcagaagaagcaggtaaaccattacctgtgaccattaatatgggtttagatccagcaatttatattggtgcttgttttgaagcaccaacaaccccttttggttataatgaattaggtgtagcaggtgctttgcgtcaaactccagtcgaattagttcagggtgtcgcagttaatgaaaaagctattgcacgtgcagaaattattattgaaggtgaactgttacctggtgtgcgtgtagaagaagatcaacatactcatacaggtcatgcaatgccagaatttcctggttattgtggtgaagcaaatccatcactgcctgtgattaaagtaaaagcagtcaccatgcgtcatcaagctattttacagactttggtgggtcctggtgaagaacatactacacttgcaggtctgccaactgaagcttctattcgtaatgctgtagaagaagcaattccaggttttcttcaaaatgtctatgctcatacagcaggtggtggtaaatttttaggtgttttgcaagtgaaaaaacgtcagccttctgatgaaggtcgtcagggtcaggcagctcttattgctctggcaacatatagcgaacttaaaaatattattctggttgatgaagatgtggatatttttgatagtgatgatattttgtgggcaatgaccactcgtatgcagggtgatgtttctattacccatcttccaggtattcgtggtcatcaattagatccaagccaggcacctgattatagtccatcaattcgtggtaatggtattacatgtaaaaccatttttgattgtacagtaccttgggcattaaaaagtcgttttgaacgtgctccatttatggaagtcgatccaaccccttgggcacctgaattgtttaaaaaataa |
| AroY_ks | atgagtaattcagaaaataaaaatacaagtggtgttaccgatctgcgttcagctattgaactgttgaaaactctgcctggtgaatatgtcgaaaccgatactgaagttgatcctcatgctgaattatctggtgtgtatcgttatgtaggtgcaggtggtacttgtcaacgtccaacacgtaaaaatggtcctgtgatgatgtttaataaagtaaaaggttttcaggatattagtgttgctattggtctgaatggttctcgtaaacgtgtgagccattttcttaattgtgcaccagaaaaattgggtcatcttctgaaagatagtgtccaaaatcctattccacctgttttaaccaaagataatgcagtgtgtcaacaggttgtgcatcttgcttcagatgcaaattttgatctgcgtaaactgttgccagctcctacaaataccgaagaagatgcaggtccatatattaccatgggtttgtgttatgcatctgatcctgaaacacatgaaagcgatattaccattcatcgtctttgtgtgcaaagtcgtgatgaactttcaatgtggctgactccaggtcgtcatattgatgcttttcgtatgaaagcagaagcagctggtcagccattgcctatttctattagcattggtgtagatccagctattgaaattgcagcttgttttgaaccacctacaacccctttaggttatgatgaattgtcagttgcaggtgctttacgtggtaaagcagttgaaatggtgcaatgtaaaacaattaatgaacgtgctattgcacatgcagaaattgtaattgaaggtgaacttctgccaaatgtccgtttgcgtgaagatcagaatactaataccggtaaagctatgccagaatttcctggttatactggtgaagctaaagatgcacttcctgtaattaaagtcaaagcagttacacatcgttataatccaatttggcgtactacagtaggtcctggtgaagaacatgtcaatatggctggtattccaaccgaagcatcaattttagatatggtgggtcgtgcaatgcctggtaaattattgaatgtctttgctcatagtgcaggtggtggtaaacttctggctgttatgcaatttaaaaaatttagcccagcagatgaaggtcgtcaacgtcaggcagctttattggctttttctgcatttcctgaacttaaacatgtgattctggtagatgaagatgttgatatttttgattctgatgatgtgctgtgggcaatgcaaacacgttatcagggtgatgtcgataccgttactattccaggtgtgcgttgtcatccattagatcctagtcagattccagcatatagtccttcaattttacaacagggcatgtcatgtaaaaccttgtttgattgtactgtaccatttcatcttaaatctcattttgaacgtagcaaatttaaagaagtagatgtcaaacgttttcttcctgattttgaataa |
| AroY_au | atgacccatgcaccattaattcgtgatttgcgtgatgcacttgatttattggcaggtgatgctgaacaacttgttgtgactgattatccagtagatcctgaagcagaattagctggtgtttataaacgtgtgggtgcaggtggtacagtggcacgtccaaccaaacgtggtcctgcaatgttatttagttcagtaaaaggttatccacgtagtcgtgtacttgtcggtctgatggcagatcgtgctcgtgtggctcgtcttctgggttgtgctccagatgcattgggtcaacgtatgtcacaggcacttgatgatgctgtagcacctgtagtcttagatgcagcagcagttgcagcagatccaagtttggctccagcacaagaagaagtttatcttgcatcagatccagattttgatattcgtcgtttattgccagctcctactaatacaccagtggatgcaggtccttatttttgtttgggtcttctgcatggttctgatccagataccggtcatagcgatgtaactattcatcgtatttgtgtccaaggtcgtgatgaattatctattttctttgctcctggtcgtcatattgatgcatttcgtgctaaagcagaagcagcaggtcaaccactgcctgttaccattaatatgggtttagatccagcaattactattggtgcttgttttgaagcaccaacaaccccttttggttatgatgaactgcaaattgcaggtggtttacgtggtcatgctgtagaactggtcccagcacgtactgtagatcagttagctattgcacgtgctgaaattgttattgaaggtgaaattttacctggtacacgtgttgtggaagatcgtaataccggtactggtcatgcaatgccagaatttcctggttatgatggtccagcaaatccttgtttaccattgatgcgtgttacagcagtgactacacgtcatcatccagttttacataccttggtgggtcctggtgaagaacatacaagtttggcaggtattccaacagaagcttcaattcttcatgcactggatgctgcattacctggtttggtaacacaagtctatgctcatacagcaggtggtggtaaactgattgcagttttacaagtggctaaacgttctgcattagatgatggtcgtgcacgtcaggctgcacttgttgcactggctacttatcatgaattgaaaaatgtaattcttgtcagccaggatgttgatgtgtttgatacagatgatgttttgtgggcaatgaccactcgtatggtgggtgatcttgatattattaccattccaggtgtatgtggtcatgttttagatccatctcaacagccagcttatgatcctcgtttgagcgcaaaaggcatgacaaccaaaactatttatgatgctacatatccttgggcaatgcgtgaaacctttgaacgtgcacaatttttcgatgttgatccagctccttggttagcagcaggtgcaccttatgaaccagatggtcaggaataa |
| AGDC1 | atgacaaccagttatgaaccatggcctcagttatattcacatttgaatggtacaaatgaagaagtgcttgatcgtatgaaagtagcagaactgtgtaaaggttggtctgtgtatcgtgatgctagcgaatgggcaaattttaaagaaatgtttaccccagatgctaatatttggactacatggtctggtgcacagactattgatagttttattcaaatttcaaaagatggtaaagataaaggtgcttttattatgcatcgtgaatgtggtacattagtcgatttgaatccaaaaacccagcgtgctattggtaaaatgaaaaccactattacacaacgttttgaatatgaaggtgttccttttgatattgattgtgataattattttattttcttttgtctgaaagattctaatggtgattggaaagctcgttggtataaagtcttttatgttaaagataaatttgttccagtgggtgtacctaccgctgaaaatatggaaaaacttgcaaaactgtttagcaaagaaaatttggaacaatatccttggggttatcagtatcttgcagttgctcaagcaaatttaggttatcctattgataaaaaattacctacttggaaaaatgaattgtatcatacaatgtatgatgcaatgaaagaatggatggagggtaaagaaattgatttacattggtaa |
| CatA* | atggaagtaaaaatttttaatactcaagatgttcaagattttcttcgtgtggcaagtggtttagaacaagaaggtggtaatccacgtgtaaaacaaattattcatcgtgttttatctgatctgtataaagctattgaagatctgaatattacaagtgatgaatattgggcaggtgtggcatatctgaatcaattaggtgcaaatcaagaagcaggtttattatcacctggtttaggttttgatcattatttagatatgcgtatggatgcagaagatgcagcattaggtattgaaaatgcaactccacgtaccattgaaggtcctttatatgtggcaggtgcaccagaaagtgtaggttatgcacgtatggatgatggttcagatcctaatggtcataccctgattttacatggtactatttttgatgcagatggtaaaccattacctaatgcaaaagtggaaatttggcatgcaaatactaaaggtttttattcacattttgatccaacaggtgaacaacaagcatttaatatgcgtcgttctattattaccgatgaaaatggtcaatatcgtgtacgtactattttaccagcaggttatggttgtccacctgaaggtcctacccaacaattactgaatcaattaggtcgtcatggtaatcgtccagcacatattcattattttgtaagtgcagatggtcatcgtaaattgacaacccaaattaatgttgcaggtgatccttatacctatgatgattttgcttatgcaacccgtgaaggtttagttgtggatgcagttgaacatacagatccagaagcaattaaagcaaatgatgttgaaggtccttttgcagaaatggtgtttgatctgaaattgacccgtttagtggatggtgtagataatcaagtagttgatcgtcctcgtttagcagtgtaa |

**Table S4.** Similar amino acid sequences to a PCA-decarboxylase AroY-kp from *Klebsiella pneumonie* were obtained by Blast search. Candidates shown in white are putative decarboxylases obtained by search and are automatically annotated as PCADC from whole genome sequences. BLAST search: *Klebsiella* and *Enterobacter* excluded, annotation was added by the NCBI Prokaryotic Genome Annotation Pipeline (released 2013), Derived by automated computational analysis using gene prediction method: Protein Homology. Candidates shown in yellow are known PCADC (characterized to catalyze PCA to catechol). Coloring of the table is used to indicate amino acid similarity between two sequences. Dark green = 80-100%, light green = 60-80%, red < 60% similarity.

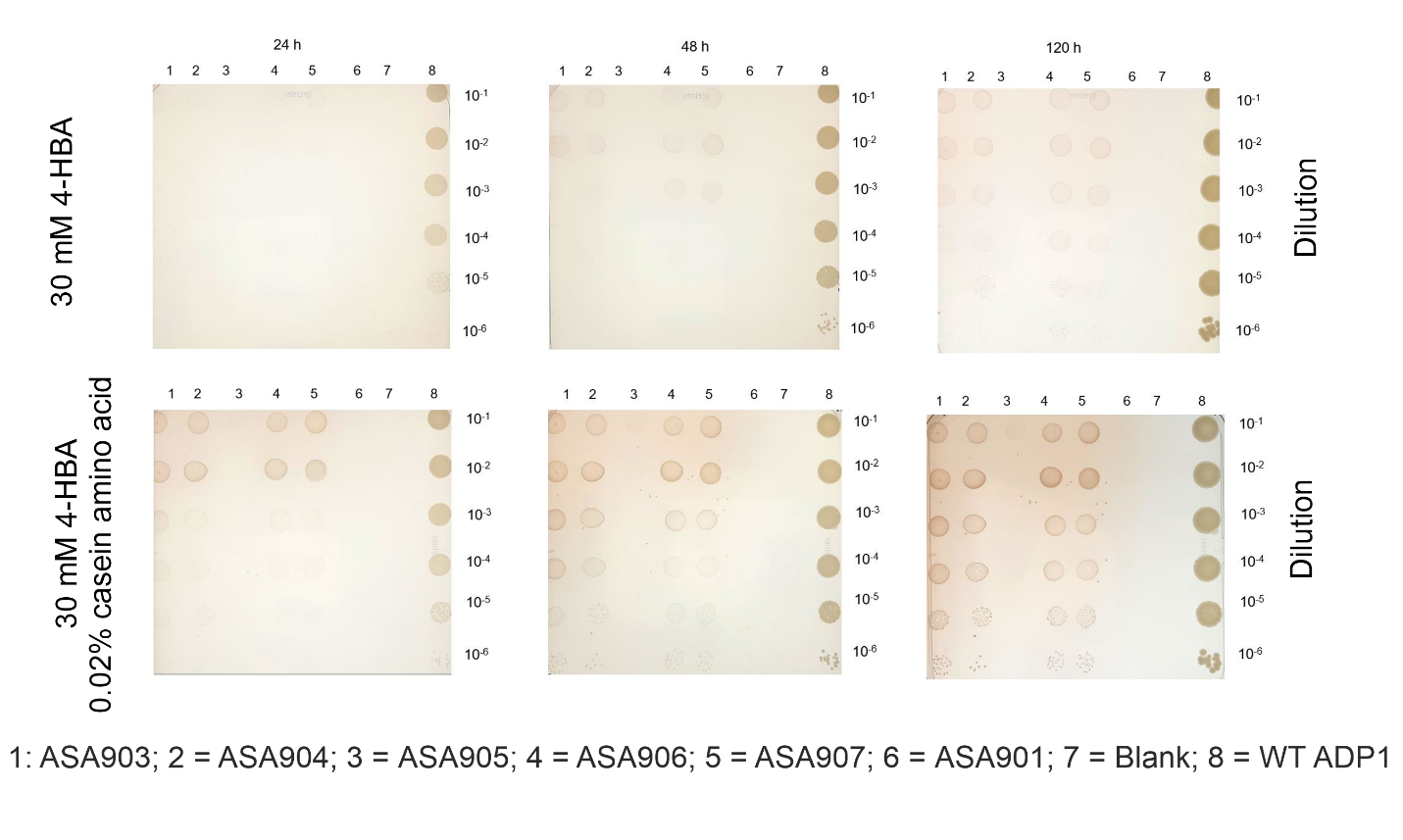


**Figure S2.** Growth-coupled selection of protocatechuate decarboxylase on solid medium plates. Precultures were grown in 5 mL of MSM supplemented with 25 mM acetate, 0.1 mM 4-hydroxybenzoate (4-HBA), 0.2% (w/v) casein amino acids, and either 25 µg/mL chloramphenicol or 30 µg/mL kanamycin, as appropriate. Cells were washed twice and resuspended in 1 mL of fresh MSM to a final OD_600_ of 1. Serial 10-fold dilutions (10^-1^to 10^-6^) were prepared, and 10 µL of each dilution was spotted onto MSM agar plates supplemented with carbon sources either 30 mM 4-HBA alone or 30 mM 4-HBA plus 0.02% (w/v) casein amino acids. Plates were incubated at 30 °C and monitored at 24 h, 48 h, and 120 h.


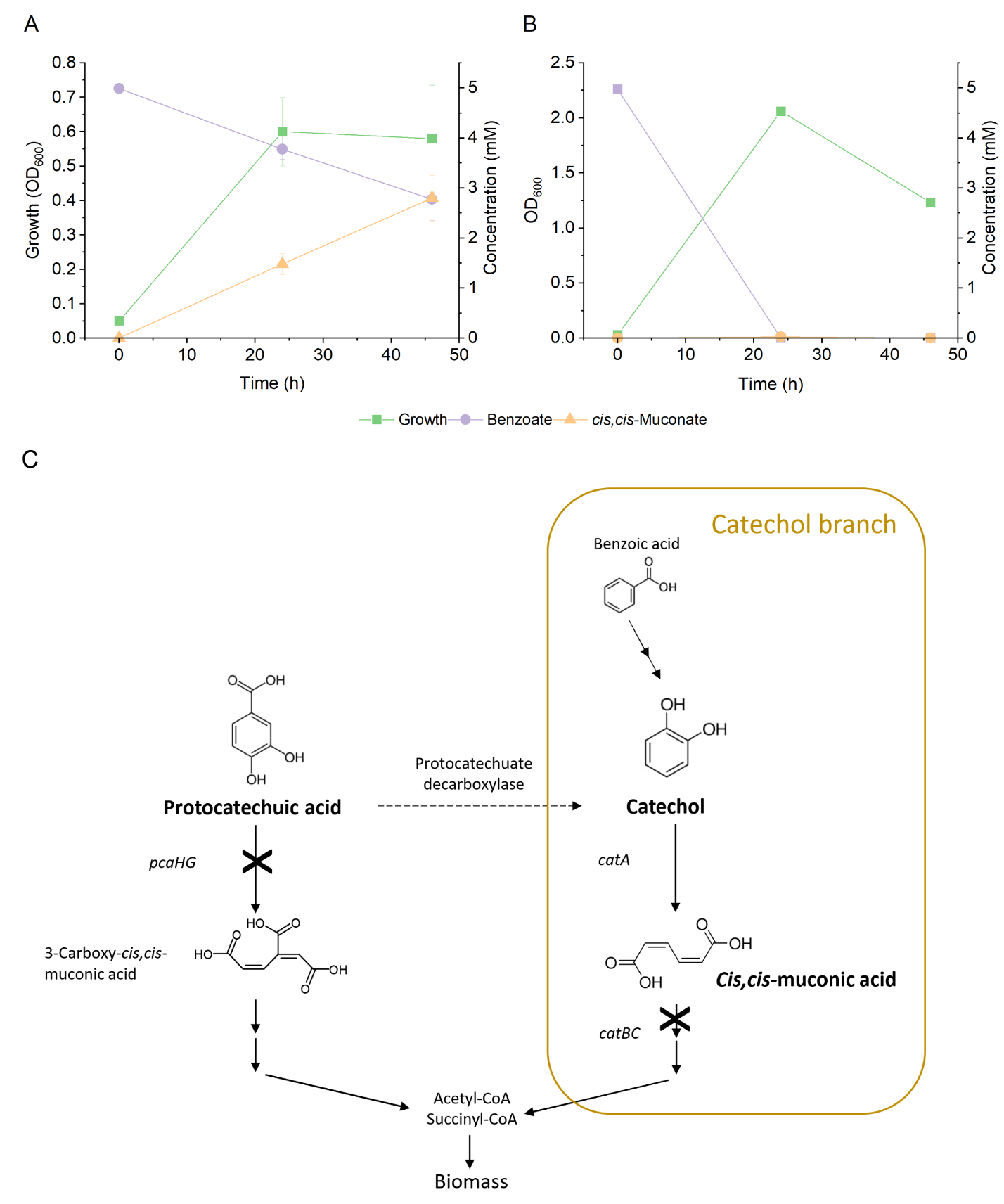


**Figure S3.** Growth profile, and concentrations of benzoate and *cis,cis*-muconate of (A) ADP1 Δ*poxB*::*aroY_au_cm^R^*; Δ*pcaH,G*; Δ*catBC::tdk/kan* and (B) ADP1 Δ*poxB*::*aroY_au_cm^R^*; Δ*pcaH,G*. (C) A schematic of the benzoate-to- *cis,cis*-muconate conversion pathway. An overnight preculture was carried out in 5 mL MSM supplemented with 5 mM benzoate, 10 mM acetate and 0.2% (w/v) casein amino acids in 14 mL culture tubes. The cultivations were carried out using 14 mL cultivation tubes with MSM supplemented with 5 mM benzoate, 10 mM acetate and 0.2% (w/v) casein amino acids for 42 hours, 30 °C, 300 rpm. The experiment was repeated using independent biological triplicates, and the averages of the measurements, with error bars representing standard deviations are shown. Error bars are present in (B) but too small to be visible.





**Figure S4.** Growth profile, cultivation volume (A), and concentrations of protocatechuate, *cis,cis*-muconate, and 4-hydroxybenzoate (B). The fed-batch cultivation was carried out by a 250 mL mini bioreactor (Applikon Biotechnology, Netherlands). The cells were first precultivated in 5 mL MSM supplemented with 0.1 mM 4-hydroxybenzoate, 0.2% (w/v) casein amino acids, and 25 mM acetate in 14 mL culture tubes at 30 °C and 300 rpm for overnight. The cells were collected and resuspended into 50 mL 5 mM 4-hydroxybenzoate, 0.2% (w/v) casein amino acids, and 25 µg/mL chloramphenicol, and transferred to the bioreactor. The cultivations were carried out at 30 °C and 200 rpm mixing, and the culture pH was maintained at 7.0 using 5 M H_3_PO_4_. MSM supplemented with 2.3 mM acetate and 25 µg/mL chloramphenicol were fed to the bioreactor with flow rate 2.8 mL/h.


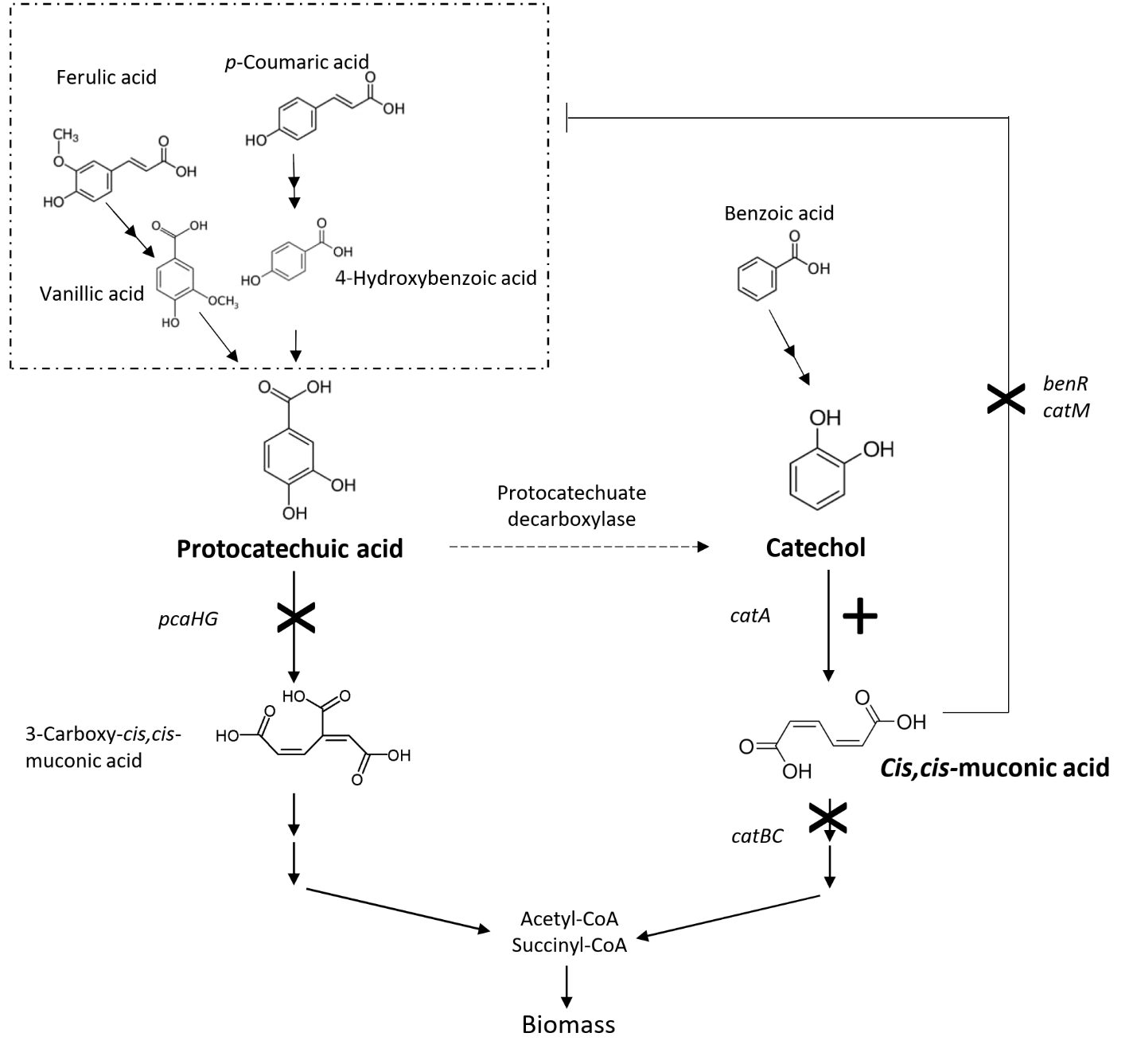


**Figure S5.** Engineered *β*-ketoadipate pathway for the production of ccMA in ADP1. Arrow with solid line indicates native pathway; arrow with dash line indicates heterologous activity; X indicated pathway blocked by the gene deletions; + symbol indicated the overexpression.





**Figure S6.** High pressure vessel for depolymerization (A) and HPLC profiles (B) of ferulate, *p*-coumarate, lignin hydrolysate, lignin hydrolysate after cultivation, wheat straw hydrolysate, and wheat straw hydrolysate after cultivation. Wild-type *A. baylyi* ADP1 was cultivated with the hydrolysates. Overnight LB precultures were used to inoculate MSM supplemented with diluted hydrolysates. Cells grew well in 10× diluted lignin hydrolysate (OD increased from ~0.02 to ~0.35) and 5× diluted wheat straw hydrolysate (OD increased from ~0.02 to ~0.091). The original lignin hydrolysate contained approximately 2.0 mM ferulic acid and 2.2 mM *p*-coumaric acid, whereas the original wheat straw hydrolysate contained approximately 0.05 mM ferulic acid and 0.058 mM p-coumaric acid. The pretreatments of wheat straw and lignin were based on the previous published methods [4–7]. The method for analyzing aromatic compounds was as follows: cultures containing wheat straw and lignin hydrolysates were centrifuged, and the supernatants were collected for HPLC analysis. Control samples without inoculum were prepared in the same manner. Prior to HPLC analysis, the supernatants were vacuum-dried and extracted with methanol. HPLC was performed using the same method described in the Analytical Methods section for the detection of ferulic acid and *p*-coumaric acid.





**Figure S7.** Growth profile and concentrations of gluconate (A), ferulate, *p*-coumarate and intermediates (B), and muconate (C). From 0 h to 48 h, MSM supplemented with 2.5 mM *p*-coumarate, 2.5 mM ferulate, 50 mM gluconate, 0.2% (w/v) casein amino acids, 100 µM IPTG, and 30 µg/mL kanamycin was fed to the bioreactor with flow rate 3.5 mL/h. Feeding was stopped after 48 h.

**References**

1. de Berardinis V, Vallenet D, Castelli V, Besnard M, Pinet A, Cruaud C, et al. A complete collection of single-gene deletion mutants of Acinetobacter baylyi ADP1. Mol Syst Biol. 2008;4:174.

2. Biggs BW, Bedore SR, Arvay E, Huang S, Subramanian H, McIntyre EA, et al. Development of a genetic toolset for the highly engineerable and metabolically versatile Acinetobacter baylyi ADP1. Nucleic Acids Research. 2020;48:5169–82.

3. Santala S, Efimova E, Karp M, Santala V. Real-Time monitoring of intracellular wax ester metabolism. Microbial Cell Factories. 2011;10:75.

4. Karp EM, Donohoe BS, O’Brien MH, Ciesielski PN, Mittal A, Biddy MJ, et al. Alkaline Pretreatment of Corn Stover: Bench-Scale Fractionation and Stream Characterization. ACS Sustainable Chem Eng. 2014;2:1481–91.

5. Linger JG, Vardon DR, Guarnieri MT, Karp EM, Hunsinger GB, Franden MA, et al. Lignin valorization through integrated biological funneling and chemical catalysis. Proceedings of the National Academy of Sciences. 2014;111:12013–8.

6. Salvachúa D, M. Karp E, T. Nimlos C, R. Vardon D, T. Beckham G. Towards lignin consolidated bioprocessing: simultaneous lignin depolymerization and product generation by bacteria. Green Chemistry. 2015;17:4951–67.

7. Vardon DR, Franden MA, Johnson CW, Karp EM, Guarnieri MT, Linger JG, et al. Adipic acid production from lignin. Energy Environ Sci. 2015;8:617–28.
